# Licoricidin combats gastric cancer by targeting the ICMT/Ras pathway in vitro and in vivo

**DOI:** 10.3389/fphar.2022.972825

**Published:** 2022-10-13

**Authors:** Hanwei Ma, Fahong Wu, Yinliang Bai, Tianwei Wang, Shangxian Ma, Liuqing Guo, Guiyuan Liu, Guangxian Leng, Yin Kong, Youcheng Zhang

**Affiliations:** ^1^ Laboratory of Hepatic-Biliary-Pancreatic, Department of General Surgery, Lanzhou University Second Hospital, Lanzhou, Gansu, China; ^2^ Department of Pediatric Gastroenterology, Lanzhou University Second Hospital, Lanzhou, Gansu, China; ^3^ Pharmacy Department, Lanzhou University Second Hospital, Lanzhou, Gansu, China

**Keywords:** Licoricidin, gastric cancer, proteomics, ICMT, cancer therapy

## Abstract

Licoricidin, a type of isoflavonoid, is extracted from the root of *Glycyrrhiza glabra*. It has been widely proven that licoricidin possesses multiple biological activities, including anti-cancer effects and a powerful antimicrobial effect against *Helicobacter pylori* (*H. pylori*). However, the exact mechanism of licoricidin against gastric cancer remains unclear. In this study, we comprehensively explored the effects of licoricidin on MGC-803 gastric cancer cells in vitro and in vivo and further elucidated its mechanism of action. Our results revealed that licoricidin exhibited multiple anti-gastric cancer activities, including suppressing proliferation, inducing apoptosis, arresting the cell cycle in G_0_/G_1_ phase, and inhibiting the migration and invasion abilities of MGC-803 gastric cancer cells. In addition to this, a total of 5861 proteins were identified by quantitative proteomics research strategy of TMT labeling, of which 19 differential proteins (two upregulated and 17 downregulated) were screened out. Combining bioinformatics analyses and the reported roles in cancer progression of the 19 proteins, we speculated that isoprenyl carboxyl methyltransferase (ICMT) was the most likely target of licoricidin. Western blot assays and IHC assays subsequently proved that licoricidin significantly downregulated the expression of ICMT, both in MGC-803 cells and in xenograft tumors. Moreover, licoricidin effectively reduced the level of active Ras-GTP and blocked the phosphorylation of Raf and Erk, which may be involved in its anti-gastric cancer effects. In summary, we first demonstrated that licoricidin exerted favorable anti-gastric cancer activities *via* the ICMT/Ras pathway, which suggests that licoricidin, as a natural product, could be a novel candidate for the management of gastric cancer.

## Introduction

According to recent global statistical data from the International Agency for Research on Cancer (IARC), about 1.1 million new cases of gastric cancer were diagnosed in 2020, resulting in more than 768,000 deaths (accounting for 7.7% of all cancer deaths) (Sung et al., 2021). Even though the incidence and mortality rates of gastric cancer have decreased over the last 50 years, it remains the fifth most frequently diagnosed cancer and is the third leading cause of malignancy-related death worldwide (Bray et al., 2018; Ajani et al., 2022). Although conventional surgical, systemic chemotherapy, radiotherapy, immunotherapy, and targeted therapy all have been widely carried out in patients suffering from gastric cancer (Joshi and Badgwell, 2021), the therapeutic effect is often limited, and some undesirable side effects are inevitable and cannot be ignored. Therefore, the burden of gastric cancer on patients and the economy is still enormous (Thrift and El-Serag, 2020), and more attractive treatment options are urgently required.

Isoprenyl carboxyl methyltransferase (ICMT) is a unique key enzyme that is involved in Ras protein activation. It also regulates carboxyl methylation, which is the final step of the complex post-translational modification of cellular proteins (Winter-Vann and Ca Sey, 2005). Ras proteins are intracellular signal transduction regulators, which control biological processes such as cell growth, differentiation, and survival under physiological conditions. Once mutated, they may induce the development of a variety of tumors. A number of previous research studies have shown that blockade of ICMT can interrupt the active procedure of Ras, thereby triggering massive cellular malfunction and cell death of various cancer cell lines (Liu et al., 2018; Qin et al., 2018; Xu J. et al, 2019; Sun and Yan, 2021). Hence, ICMT has been considered as novel and promising tumor therapy target (Yang et al., 2017). In recent years, natural medicinal therapy, like medicinal plants and their active ingredients, has emerged as a new trend in the field of anti-tumor therapy because of its high efficiency and low side effects (Karthika and Sureshkumar, 2021). Since spermatinamine (the first natural product inhibitor of ICMT) was discovered for cancer therapy (Buchanan et al., 2007), more natural inhibitors of ICMT have been identified in succession (Bharate et al., 2012).

**FIGURE 1:**
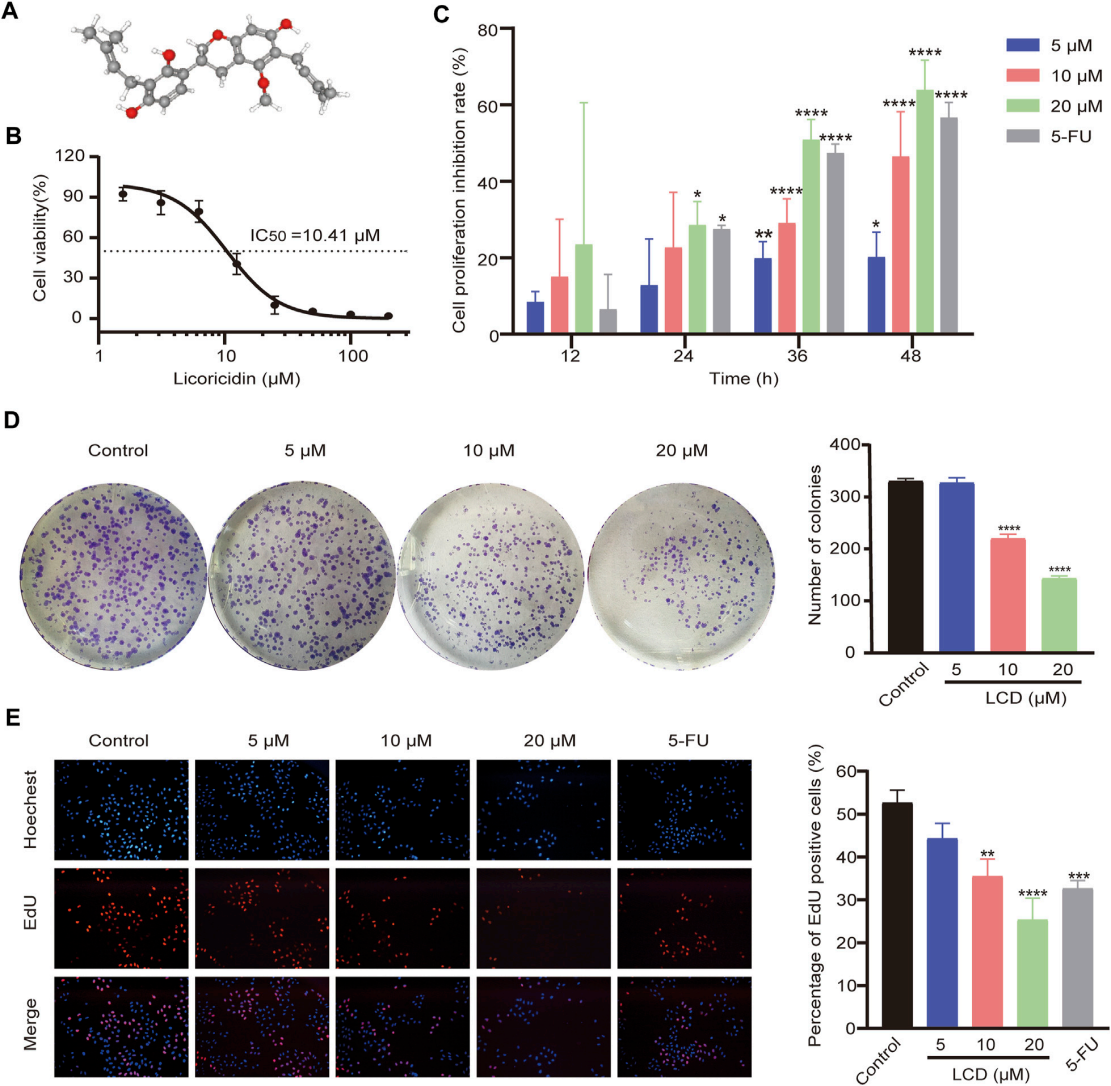
Chemical structure of licoricidin and its inhibitory effect on the proliferation of MGC-803 cells. **(A)** Three-dimensional (3D) chemical structure of licoricidin, downloaded from Pubchem (https://pubchem.ncbi.nlm.nih.gov/compound/480865). **(B)** Half-maximal inhibitory concentration (IC_50_) of licoricidin on MCG-803 cells. Cells were treated with licoricidin (1.5625, 3.125, 6.25, 12.5, 25, 50, 100, and 200 μM) for 24 h. OD values at 450 nm were detected by CCK-8 assay, and then the IC_50_ was calculated by Graphpad Prism 8.0. **(C)** Licoricidin inhibited the proliferation of MGC-803 cells *via* CCK-8 assay. Cells were administrated with licoricidin (5 μM, 10, and 20 μM) for 12, 24, 36, and 48 h. **(D)** Colony formation of MGC-803 cells treated with licoricidin. **(E)** EdU-positive cells detected by the EdU staining test (×100). MGC-803 cells were exposed to licoricidin (5 μM, 10, and 20 μM) or 5-fluorouracil 20 μM for 24 h. Results are displayed as mean ± SD of three independent experiments. *p < 0.05, **p < 0.01, ***p < 0.001, and ****p < 0.0001. LCD, licoricidin. 5-FU, 5-fluorouracil.

Licoricidin, a type of isoflavonoid (Figure 1A), was first identified and reported by Japanese scholars when they attempted to isolate chemical components from the root of *Glycyrrhiza glabra* for treatment of gastric ulcers in 1968 (Shibata and Saitoh, 1968). Since then, various biological activities of licoricidin have been gradually discovered, and its outstanding performance in tumor prevention and treatment has attracted extensive attention. For instance, it has been preliminarily proven that it exhibits versatile biological activities against prostate cancer (Park et al., 2010), breast cancer (Park et al., 2016), hepatocellular carcinoma (Cevik et al., 2019), colorectal adenocarcinoma (Ji et al., 2017), and osteosarcoma (Wang et al., 2018) and could also block DNA damage caused by carcinogenic N-methyl-N-nitrosourea (Inami et al., 2017). More importantly, licoricidin exerts a bacteriostatic effect on *H. pylori*, which is the group Ⅰ carcinogen for gastric cancer (Crowe, 2019), and its antibacterial activity against the growth of clindamycin and amoxicillin-resistant strain GP98 was still considerable: its minimum inhibitory concentration (MIC) was 6.25 μg/mL against 2 × 10^5^ cfu of this strain (Fukai et al., 2002). Nevertheless, there have been very few research studied on the roles of licoricidin in gastric cancer, not to mention the specific mechanism.

In this study, we first confirmed that licoricidin exerted an anti-tumor effect on gastric cancer in vivo and in vitro. Then, we confirmed ICMT protein as a target by the proteomics research strategy of TMT labeling and further elucidated the anti-cancer mechanism. It is of great significance to provide a research foundation for licoricidin as it can potentially be used as an anti-gastric cancer candidate molecule in the future.

## Materials and methods

### Chemicals and dilution

Licoricidin (CAS. 30508-27-1, molecular weight: 424.5, purification ≥ 98%) and 5-fluorouracil (CAS. 51-21-8, molecular weight: 130.08, specification 99%) were purchased from ChemFaces (Wuhan, China) and AIKE REAGENT (Chengdu, China), respectively. They were dissolved in dimethyl sulfoxide (Solarbio, Beijing) and then stored in the dark.

### Cell culture

The human gastric cancer cell line MGC-803 was obtained from iCell Bioscience Inc (Shanghai, China) and maintained in RPMI Medium Modified (HyClone, United States), supplemented with 10% fetal bovine serum (FBS) (Biological Industries, Israel) and 1% penicillin–streptomycin (Gibco, United States). The cells were cultured in a humidified incubator at 37°C with 5% CO_2_ and digested by 0.1% trypsin (Biological Industries, Israel) for cell passage for 1–2 days. Exponential growth cells within 10 generations were used in our experiments.

### CCK-8 assay

CCK-8 assay was used to determine the effect of licoricidin (LCD) on cell viability. MGC-803 cells at the logarithmic growth stage were counted by a cell counter (Denovix CellDrop FL, United States) and then seeded in a 96-well plate at a density of 6 × 10^3^–10^4^ cells per well. We incubated the cells at 37°C with 5% CO_2_ overnight. When the cells adhered to the wall, they were treated with different concentrations of licoricidin or 5-fluorouracil (5-FU). In addition, the control group including the culture medium only was set to control bias. The duration of the treatments depended on the experimental requirements. Specifically, the cells were treated for 24 h to calculate the half-maximal inhibitory concentration (IC_50_), while the cell proliferation inhibition tests were performed after interventions for 12, 24, 36, and 48 h. Next, 10 μL of CCK-8 enhanced solution (Meilunbio, Dalian, China) was added to each well in the dark and we then continued to incubate for 1 h. The absorbance at a wavelength of 450 nm was measured with a Mulltiskan FC microplate reader (Thermo Fisher Scientific, United States). Cell inhibition rate (%) = [(control group-experimental group) / (control group-blank group)] × 100%. Independent experiments were carried out with triplicate wells for each group and repeated three times.

### Cell colony formation assay

The influence of licoricidin (LCD) on the proliferation of MGC-803 cells was observed by the cell colony formation method. We digested the cells in the logarithmic growth phase and then added the complete medium to prepare a single-cell suspension. The cells were counted by a cell counter (Denovix CellDrop FL, United States) and then seeded in a 6-well plate (4 mL of the complete medium containing 800 suspended cells was added to each well). Cells were cultured at 37°C with 5% CO_2_, and different concentrations of LCD (5, 10, and 20 μM) were added and mixed gently with the medium when the cells adhered to the wall. The incubation was continued until 14 days later when the cell colonies could be observed with the naked eye. Subsequently, we discarded the old medium and washed twice. The colonies were fixed with 4% paraformaldehyde for 30 min and then stained with 0.5% crystal violet for 15 min. Finally, they were washed with pure water to remove excess staining solution and then dried at room temperature and photographed with an ordinary camera. The number of colonies containing at least 50 cells was counted with ImageJ software. Independent experiments were repeated three times.

### EdU assay

The logarithmically grown cells were seeded in a 96-well plate. When the cells reached up to 60% confluence on the well, they were treated with different concentrations of LCD (0, 5, 10, and 20 μM) or 5-FU (20 μM) for 24 h. According to the protocol of the Cell-Light ™ EdU Apollo567 In Vitro Kit (Ribobio, Guangzhou, China), the EdU pre-made solution was added to the wells and labeled in the incubator for 2 h of culturing. The cells were fixed with paraformaldehyde at room temperature for 0.5 h and subsequently washed. We then performed Apollo staining and Hoechst 33342 staining in sequence, which was followed by washing. Images were immediately captured by an inverted fluorescence microscope (Olympus, Japan) with identical shooting parameters. ImageJ software was used to further merge the images, and the cells were counted. The nuclei of EdU-positive cells were stained red and reflected higher cell viability. Percentage of EdU-positive cells (%) = number of EdU-positive cells/total number of cells × 100%.

### Flow cytometry assay

Different interventions (0, 5, 10, and 20 μM of LCD or 20 μM of 5-FU) were performed on the well-grown cells in a 6-well plate for 24 h. The cells were then processed according to the procedures of the corresponding kits. Apoptosis or cell cycle of MGC-803 cells was then assessed by using a CytoFLEX flow cytometer (Beckman, United States). Each test was carried out on three separate occasions. The details are provided below:

Apoptosis analysis was performed based on the steps recommended by the Annexin Ⅴ-FITC/PI Apoptosis Detection Kit (Yeasen, Shanghai, China). We collected the trypsinized cells and the old cell culture medium, which were then centrifuged at 4°C, 1500 rpm, for 5 min (the supernatant was discarded). We washed the cells with pre-cooled PBS and centrifuged them according to the previously mentioned parameters and then discarded the PBS. Next, cell concentration was adjusted to more than 1× 10^6^ cells /mL by resuspending cells with a 1× binding buffer. Meanwhile, 100 uL of the cell suspension from the different groups was transferred into homologous flow tubes. Subsequently, 5 μL of Annexin V-FITC and 10 μL of PI staining solution were added to each tube, which were then incubated in the dark for 15 min at room temperature. Finally, a 1× binding buffer was complemented to total the test volume up to 500 μL per tube. After mixing gently, we placed the tubes on ice and detected the percentage of apoptosis using a flow cytometer.

Cell cycle analysis was conducted according to the protocol of Cell cycle staining Kit guide (Multi Sciences, Hangzhou, China). We discarded the old cell culture medium. The cells were then sequentially digested with trypsin and washed twice with ice-cold PBS and centrifuged at 4°C, 1500 rpm, for 5 min (the supernatant was discarded). We then resuspended the cells with 300 μL of cold PBS in each tube, fixed the cells by slowly adding 700 μL of pre-cooled absolute ethanol, carefully flicked the tube wall to disperse the solution, and then continued to mix it. The samples were placed in a 4°C refrigerator overnight. Then, they were centrifuged and washed on the following day. Finally, we added 1 mL of DNA staining solution into each tube and left them to incubate in the dark at room temperature for 30 min after shaking and mixing well. The cell cycle was measured by flow cytometry at the lowest loading speed, and the data were analyzed by ModfitLT 5.0 software.

### Morphological study

The morphological changes of apoptotic cells were observed by Giemsa staining. The suspension of MGC-803 cells was seeded in a 24-well plate and cultured overnight. The cells were treated with 20 μM of LCD, while the untreated cells were used as the control group. After culturing for 24 h, we discarded the old cell culture medium and washed twice with PBS. Subsequently, we added methanol to fix the cells for 10 min. Finally, we removed the fixed liquor and strained with 10 × Giemsa dye solution (Solarbio, Beijing, China) for 10 min. This was followed by washing with water. The morphology of cells was observed with an inverted microscope (Olympus, Japan) and photographed.

### Transwell assay

MGC-803 cells were starved for 2 h and then collected after trypsin digestion. They were then resuspended in a serum-free medium to prepare a 1.5 × 10^5^ cell/mL cell suspension. Meanwhile, 200 μL of the cell suspension and intervention drug (0, 5, 10, and 20 μM of LCD, or 20 μM of 5-FU) were mixed and added to the upper chamber of the corresponding Transwell (Corning 3422, United States), while 600 μL of a medium containing 25% FBS was added into the lower chambers. We then placed the 24-well plate in the incubator. After 24 h, the chambers were removed and fixed with 4% paraformaldehyde for 10 min at room temperature and subsequently washed with PBS and immersed into another 24-well plate filled with 700 μL of 1% crystal violet staining solution per well. They were then dyed for 30 min at 37°C. Next, we removed the chambers again, gently wiped the cells and crystal violet in the upper layer of the chambers with cotton swabs, washed them three times with PBS, and finally air-dried them. The number of migrated/invaded cells was observed under a microscope (×200), and a total of five fields of view were counted. The chambers of the invasion experiments needed to be coated with Matrigel matrix (Corning 356234, United States) in advance, that is, we mixed the matrix and serum-free medium at a ratio of 1:8 and added them into the upper chambers and then placed them in a cell incubator for 3–4 h. Then, we dislodged the supernatant gently and added 70 μL of serum-free medium to each chamber, placed the chambers again in the incubator for 1 h, and discarded the supernatant for later use. The other processing steps were identical to those of the migration experiment.

### Wound healing experiment

MGC-803 cells with a good cellular growth behavior were seeded in a 6-well plate and cultured overnight. When the density of cells reached up to more than 90%, the monolayer in each well was scratched using a sterilized 10-uL pipette tip and an ultraviolet irradiated ruler and then washed with PBS three times. We then added 100 uL medium containing 2% FBS to each well and photographed the scratches. Next, the medium containing 2% FBS and the indicated concentration of LCD (0, 5, 10, and 20 μM) or 20 μM of 5-FU were added to the corresponding wells. After incubating for 24 h, the scratches were photographed again. Independent experiments were repeated three times. The wound areas were outlined and measured with ImageJ software. Wound healing rate (%) = (wound area at 0 h -wound area at 24 h)/wound area at 0 h × 100%.

### Xenograft nude mice model for experiments in vivo

Male nude mice (BALB/c-Nu), 5 weeks old, 20 ± 2 g, were purchased from Gempharmatech Co., Ltd. (Nanjing, China) [License number: SCXK (Su) 2018-0008]. The animal experiment protocol was approved by the Laboratory Animal Welfare Ethics Committee of the Second Hospital of Lanzhou University (D2021-114). All procedures were in accordance with the Laboratory Animal—Guideline for ethical review of animal welfare (GB/T35892-2018), which is jointly issued by the General Administration of Quality Supervision, Inspection and Quarantine and Standardization Administration Committee (China). All animals were grown under the safety environmental conditions provided by the SPF animal laboratory of Lanzhou University Medical Experiment Center.

Cells in the logarithmic growth phase were collected and counted. We then adjusted the final concentration of the cell suspension to 10^7^ cells /mL with PBS. We mixed the cell suspension and the pre-thawed Matrigel at a ratio of 1:2. 0.2 mL of the tumor-forming solution was subcutaneously injected into the left armpit of each of the nude mice. Thereafter, the tumor diameters were measured with a vernier caliper, and the tumor volume (V) was estimated using the formula V = A × B^2^/2 (where A was the largest diameter and B was the smallest diameter). When the tumor volume reached 50–100 mm^3^, the mice were randomly divided into four groups (*n* = 6), and we intraperitoneally injected the corresponding drugs once every 2 days, including 10 mg/kg of LCD, 20 mg/kg of LCD, 20 mg/kg of 5-FU, or equal volumes of normal saline in the control group mice. The body weight and tumor volume of nude mice were recorded every 3 days during the treatment. The mice were euthanized 3 days after the last injection. The tumors were weighed and then frozen in reserve.

### Western blot assay

The cells were washed three times with pre-cooled PBS and lysed on ice for 30 min with lysis buffer containing RIPA, PMSF, phosphorylated protease inhibitor, and cocktail protease inhibitor (Sevier, Wuhan). The cells were then scraped and collected into Eppendorf tubes and centrifuged at 12,000 rpm for 15 min at 4°C. To extract the proteins in the tumor tissues, we needed to break the tissues quickly with a homogenizer in advance. Subsequently, the broken tissues were lysed on ice for 30 min and centrifuged (the parameters were identical to those previously mentioned). About 200 uL of lysate buffer (same formula as before) was required per 100 mg of tissue. The supernatant of proteins obtained by centrifugation was added into 5 × SDS-PAGE protein loading buffer (Solarbio, Beijing, China), boiled in water for 15 min, and then stored at −20°C. The concentration of proteins was determined using the BCA Protein Assay Kit (Solarbio, Beijing, China).

Different concentrations of sodium dodecyl sulfate (SDS)-polyacrylamide gel were prepared according to the protocol of the SDS-PAGE Gel Kit (Solarbio, Beijing, China). The lysed proteins (30 μg) were resolved on the gels and electro-transferred onto polyvinylidene difluoride (PVDF) membranes (Biosharp, Hefei, China). The membranes were blocked with 5% milk (Solarbio, Beijing, China), dissolved in the Tris-buffered saline for 2 h at room temperature, and then blotted with primary antibodies at 4°C overnight. The primary rabbit antibodies used in this study included Bcl-2 (AF6139, Affinity, Jiangsu, China), Bax (AF0120), Cyt-C (AF0146), MMP9 (AF5228), Ras (AF0247), p-Raf (AF3065), p-ERK (AF1015), β-actin (AF7018), β-tubulin (AF7011), GAPDH (AF7021), Ras-GTP (DF2645, Affinity), MMP2 (BM4075, Boster, Wuhan, China), CDK4 (BM4672), cyclin D1 (BA0770-2, Boster), Caspase 3 (19677-1-AP, Proteintech, Wuhan, China), and ICMT (GTX129471, GeneTex, United States). On the next day, the membranes were incubated with horseradish peroxidase (HRP)-conjugated secondary antibody (A21020, Abbkine, Wuhan, China) for 2 h at room temperature. Next, we washed the membranes with TBST and then added the SuperKine™ West Femto Maximum Sensitivity Substrate (Abbkine, Wuhan, China) to the membranes. Finally, the blots were scanned using an automatic chemiluminescence image processing system (Tanon 5200 Multi, Shanghai, China). The gray values of immunoreactivity for each protein were analyzed by ImageJ software.

### Immunohistochemical method

Tumor tissues soaked in 4% paraformaldehyde were sequentially dehydrated, dipped in wax, embedded, and then sliced. Afterward, the paraffin sections were degreased with xylene, deparaffinized with ethanol *via* a decreasing concentration gradient, and washed with distilled water. We placed the sections in a pressure cooker, which was filled with citric acid solution (pH 6.0) for 2.5 min to repair the antigen. We then placed them in a 10% hydrogen peroxide solution, and they were incubated at room temperature for 20 min to block endogenous peroxidase. Next, 5% BSA was added dropwise over the area of the delineated tissue and blocked at room temperature for 1 h. Primary antibodies with different concentrations (Bcl-2, Bax, and ICMT, 1:100, Ki-67, 1:400) were then added and incubated at 4°C overnight. The next day, the secondary antibody was added and incubated at room temperature for 50 min. A DAB chromogenic solution was added dropwise until the sections turned yellow. Finally, the slices were counterstained with Harris hematoxylin, dehydrated, and subsequently sealed with mounting medium in sequence. Images (×200) were observed and photographed with the TissueFAXS Plus panoramic tissue and a cell quantification system (TissueGnostics, Austria). The expression levels of the proteins were analyzed quantitatively using Image-Pro Plus software.

### Proteomics analysis

The experiments consisted of a control group and an LCD group (treated with 10 μM of LCD for 24 h). Each group consisted of three replicate samples, a total of six samples, and the number of cells in each sample reached (2-10) × 10^7^. Proteins of the samples were extracted by lysing the cells with an SDT (4% SDS, 100 mM Tris-HCl, 1 mM DTT, pH 7.6) buffer and quantified with the BCA Protein Assay Kit (Bio-Rad, United States). Proteins were digested into peptides with trypsin considering the filter-aided sample preparation (FASP) course. The digested peptides were then desalted on C18 Cartridges (Empore™ SPE Cartridges C18 (standard density), bed I.D. 7 mm, volume 3 mL, Sigma), concentrated by vacuum centrifugation, and reconstituted in 40 µL of 0.1% (v/v) formic acid. We estimated the contents with UV light spectral density at 280 nm. According to the protocol (Thermo Scientific), 100 μg peptide mixture of each sample was labeled (LCD-1 ^TMT126^, LCD-2^TMT127^ LCD-3 ^TMT128^, Control-1 ^TMT129^, Control-2 ^TMT130^, Control-3 ^TMT131^) with TMT reagent and fractionated using SCX chromatography using the AKTA purifier system (GE Healthcare). LC-MS/MS analysis was conducted with a Q-Exactive mass spectrometer (Thermo Scientific), which was coupled to a HPLC liquid system Easy nLC (Thermo Fisher Scientific), whose nanoliter flow rates were 60/90 min. The original data of MS were RAW files and embedded in Proteome Discoverer 1.4 software for identification and quantitative analysis using the MASCOT engine 2.2 (MATRIX Science, London, United Kingdom). Differentially expressed proteins were screened out based on the Fold Change (FC) expression over 1.2 times (i.e., upregulation greater than 1.2 times or downregulation less than 0.83 times) and a *p* value <0.05 (paired Student’s *t*-test).

### Bioinformatics analysis

Hierarchical clustering analysis was carried out using Cluster 3.0 and Java Treeview software. A multi-class SVM classification system, named CELLO (http://cello.life.nctu.edu.tw/), was used to speculate the subcellular location of proteins. Pfam database, Blast2GO, and KAAS (KEGG Automatic Annotation Server) software were used to annotate the corresponding biological information of the domain, GO pathway, and KEGG pathway, respectively. Moreover, the enrichment analyses of these annotations were performed by Fisher’s exact test.

### Molecular docking study

Schrodinger software (issue 2019-2, Schrödinger, LLC, New York, NY2019) was used to study the molecular docking model of licoricidin and ICMT. The protein structure of ICMT (Diver et al., 2018) was preprocessed and optimized with the protein preparation wizard tool in Schrodinger software. Chembiodraw Ultra 12.0 software was used to construct the molecular structure of licoricidin and produce its 3D coordinates. Molecular docking was manipulated with extra precision (XP) after generating a 10-Å receptor grid. The other docking parameters remained at their default values.

### Statistical analysis

All data are expressed as mean values ± standard deviation (SD). Statistical analyses of the differences between two groups were performed using one-way analysis of variance (ANOVA) and subsequently by unpaired Student’s *t*-test. The differences were statistically significant at the *p* values <0.05.

## Results

### Licoricidin suppressed the growth of MGC-803 cells *via* inhibiting proliferation in vitro

Cells were treated with different concentrations (200, 100, 50, 25, 12.5, 6.25, 3.125, and 1.5625 μM) of licoricidin for 24 h. The IC_50_ of licoricidin was then calculated as 10.41 μM (Figure 1B). The intervention concentrations of licoricidin (5, 10, and 20 μM) in this study were chosen according to the IC_50_. The 5-fluorouracil group was added as a positive control. The cells were treated with corresponding concentrations for 12, 24, 36, and 48 h, and their viabilities were reflected by OD values at 450 nm. As shown in Figure 1C, licoricidin inhibited the growth of MGC-803 cells in a time- and dose-dependent manner, and the difference was statistically significant. Next, cells were treated with drugs of indicated concentrations for 24 h. As shown in Figure 1D, the numbers of formed cell colonies were significantly lower than those of the control group at 10 and 20 μM of licoricidin. In addition, the rate of EdU-positive cells remarkably reduced in a dose-dependent manner (Figure 1E). Collectively, these results revealed that licoricidin suppressed proliferation of MGC-803 cells in vitro.

### Licoricidin induced apoptosis of MGC-803 cells and arrested mitosis in the G_0_/G_1_ phase

**FIGURE 2:**
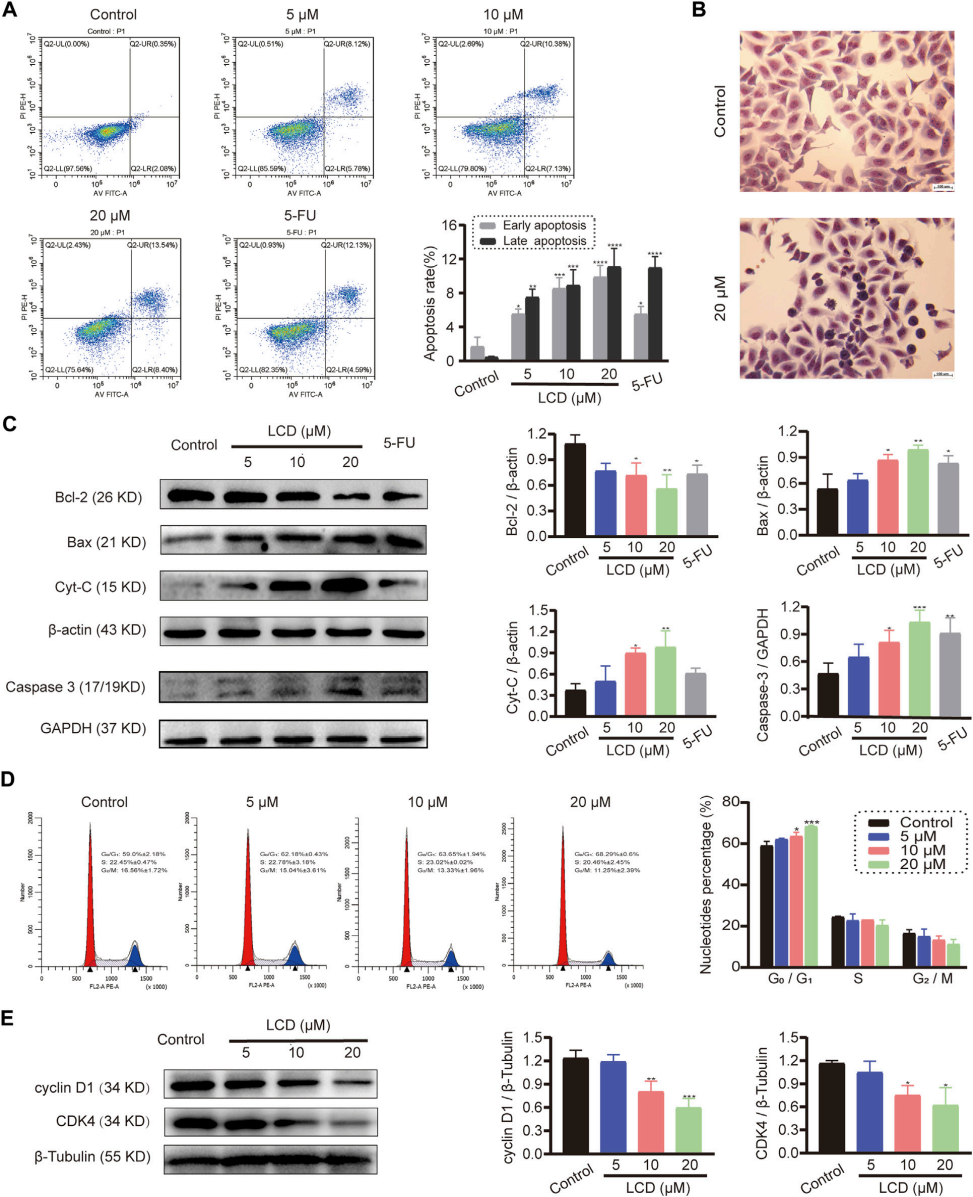
Licoricidin promoted MGC-803 cell apoptosis and arrested mitosis in the G_0_/G_1_ phase. MGC-803 cells were treated with licoricidin (5, 10, and 20 μM) or 5-fluorouracil 20 μM for 24 h. **(A)** Apoptosis of MGC-803 cells was detected by flow cytometry after Annexin Ⅴ-FITC/PI dual staining, and the percentage rate of apoptotic cells was displayed in the histogram. **(B)** Morphological changes of MGC-803 cells were observed by Giemsa dye liquor (×200). Compared with the untreated group, cells treated with 20 μM of licoricidin presented a different morphology, and particularly, typical manifestations of apoptotic cells, such as nuclear pyknosis, hyperchromasia, and apoptotic bodies, can be seen. **(C)** Expression levels of apoptosis-related proteins (Bcl-2, Bax, Cyt-C, and Caspase 3) were assessed by Western blot assay and normalized by reference genes for quantitative analysis. **(D)** Licoricidin arrested the cell cycle in the G_0_/G_1_ phase. Flow cytometry was carried out to measure DNA content and cell cycle distribution after the cells were fixed by PI staining, and the results were analyzed by ModfitLT 5.0 software. **(E)** Expression levels of cyclin D1 and CDK4 were assessed by Western blot analysis and normalized by β-tubulin for quantitative analysis. Results are displayed as mean ± SD of three independent experiments. *p < 0.05, **p < 0.01, ***p < 0.001, and ****p < 0.0001. LCD, licoricidin. 5-FU, 5-fluorouracil. Scale bar, 100 μm.

To explore the effect of licoricidin on apoptosis in vitro, we performed Annexin V-FITC/PI dual staining of MGC-803 cells using flow cytometry and Giemsa dye assay and Western blot analysis against marker proteins of apoptosis (Bcl-2, Bax, Cyt-C, and Caspase 3). Within the tested dose range, the Annexin V-FITC/PI staining assay showed that the percentage of apoptotic cells increased in a concentration-dependent manner (Figure 2A). Next, the cells treated with 20 μM of licoricidin presented different morphology using Giemsa staining. In particular, typical manifestations of apoptotic cells (Xu X. et al, 2019)—such as nuclear pyknosis, hyperchromasia, and apoptotic bodies—could be observed in the licoricidin-treated group (Figure 2B). Finally, Western blot analysis indicated that the expression level of Bcl-2 protein in MGC-803 cells clearly decreased, while the expressions of Bax, Cyt-C, and Caspase 3 increased gradually along with the increasing doses of licoricidin (Figure 2C). On the other hand, the changes in the cell cycle pattern of licoricidin-treated MGC-803 cells were also analyzed by flow cytometry assay. Compared to the control group, the nucleotide percentages of the G_0_/G_1_ phase were significantly increased with a higher dose of licoricidin (10 and 20 μM), as shown in Figure 2D. Moreover, the corresponding representative cell cycle-regulating proteins, cyclin D1 and CDK4, were significantly reduced (Figure 2E). Together, licoricidin promoted apoptosis of MGC-803 cells and arrested cell mitosis in the G_0_/G_1_ phase in a concentration-dependent manner.

### Licoricidin inhibited the migration and invasion of MGC-803 cells

**FIGURE 3:**
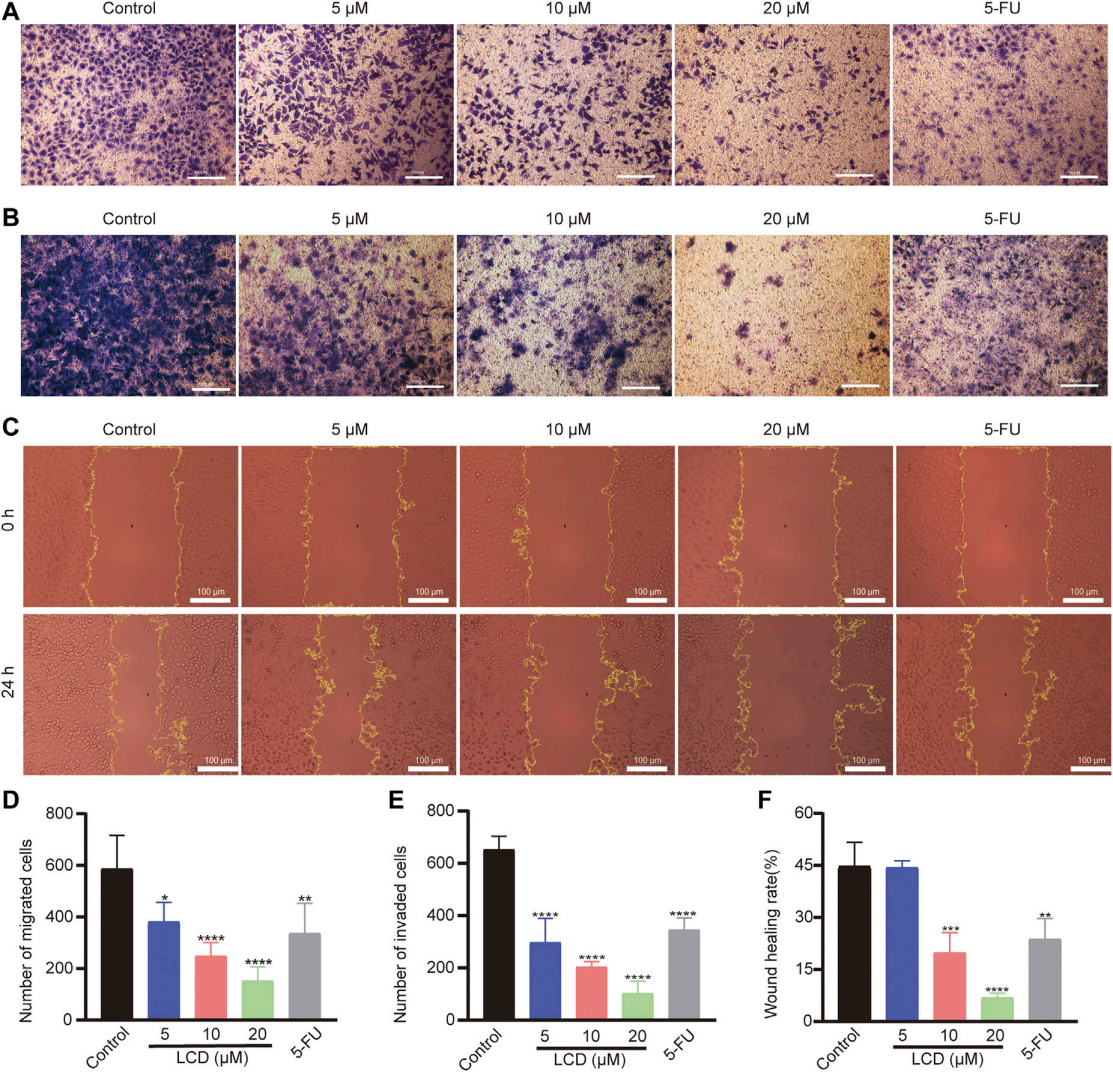
Licoricidin suppressed MGC-803 cell metastasis. MGC-803 cells were treated with licoricidin (5, 10, and 20 μM) or 5-fluorouracil 20 μM for 24 h. Representative images of cell migration **(A)** and invasion **(B)** in each group were detected by transwell experiments (×200). The number of migrated **(D)** or invaded **(E)** cells passing through the membrane in each well was counted from five random fields of view and analyzed as the mean ± SD. **(C,F)** Comparison of wound healing areas of MGC-803 cells treated with indicated concentration for 24 h (×200). The parts circled in yellow lines represent areas where the cells had not yet migrated to fill. **(G)** Expression levels of MMP2 and MMP9 were assessed by Western blot analysis and normalized by β-actin for quantitative analysis. Results are displayed as mean ± SD of three independent experiments. *p < 0.05, **p < 0.01, ***p < 0.001, and ****p < 0.0001. LCD, licoricidin. 5-FU, 5-fluorouracil. Scale bar, 100 μm.

To discover the effect of licoricidin on the motility ability of MGC-803 cells, the transwell invasion / migration assay, wound healing assay, and Western blot analysis were all carried out after cells were treated with licoricidin for 24 h. As shown in the respective images (Figures 3A,B), the numbers of migration/invasion of cells passing through the transwell membranes significantly reduced in each treatment group in a dose-dependent manner (Figures 3D,E). Next, the wound healing status of MGC-803 cells, with or without the intervention of licoricidin, was observed. Compared with the control group, the higher the concentration of licoricidin, the lower the wound-healing rate of the cells displayed. Furthermore, the wound area of the high-dose group (20 μM of licoricidin) was almost unchanged after the treatment (Figures 3C,F). In addition, Western blot results also showed that the secretion of MMP2 and MMP9 decreased remarkably in a concentration-dependent manner (Figure 3G). In summary, these data indicate that licoricidin could weaken the migration and invasion capacities of MGC-803 cells.

### Licoricidin prevented MGC-803 cell xenografted tumor growth in vivo

**FIGURE 4:**
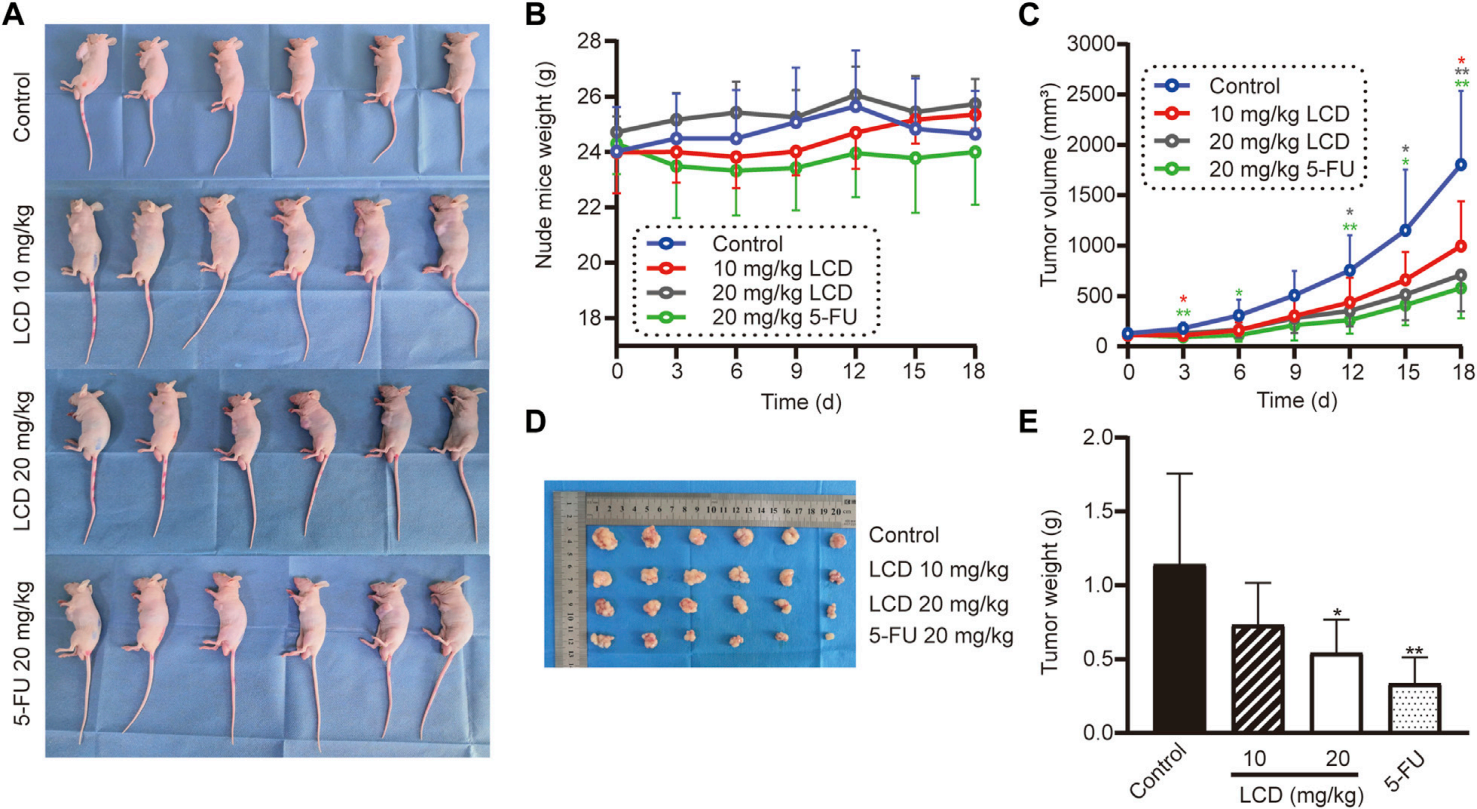
Licoricidin prevented MGC-803 cell xenografted tumor growth in vivo. **(A)** Models of the MGC-803 cell xenografted tumor in nude mice, divided into four groups (*n* = 6). After the xenografted tumor models were established successfully, we administered an intraperitoneal injection of normal saline, licoricidin 10 mg/kg, licoricidin 20 mg/kg, or 5-fluorouracil 20 mg/kg once every 2 days in the corresponding groups. Nude mouse weight **(B)** and tumor volume **(C)** were measured once every 3 days. The course of treatment was 15 days in total, and then all mice were euthanized after 3 days. **(D)** Macroscopic appearance of xenografted tumors. **(E)** Tumors were weighed after they were removed from the bodies. Values are expressed as mean ± SD, *p < 0.05 and **p < 0.01. LCD, licoricidin. 5-FU, 5-fluorouracil.

According to the abovementioned experiment results *in vitro*, we predicted that licoricidin could be a promising agent against gastric cancer. We then evaluated finally whether licoricidin also inhibited tumor growth in vivo based on the xenografted tumor model. MGC-803 cells were subcutaneously inoculated into the left armpit of nude mice (10^7^ cells/mouse). When the tumor volume of each mouse reached 50–100 mm^3^, the mice were randomly divided into four groups and were intra-peritoneally injected the indicated agents, once every 2 days. We euthanized the mice on the 18th day (Figure 4A). The body weight and tumor volume of nude mice were recorded every 3 days during the treatment. The measurement results showed that the weight of the nude mice was essentially stable during the observation period (Figure 4B). Compared with the control group, the amplification of the tumor volume in each of the treated groups gradually slowed down. Furthermore, when treated with a higher dose of licoricidin, lesser tumor volume at the same detection time point was observed (Figures 4C,D). It should be noted that the 5-fluorouracil-treated group exhibited stronger anti-cancer activity than the high-dose licoricidin group (20 mg/kg). In addition, the difference between the two groups was not statistically significant (Figure 4E). Concisely, it was elementarily manifested that licoricidin could block tumor growth in vivo.

**FIGURE 5:**
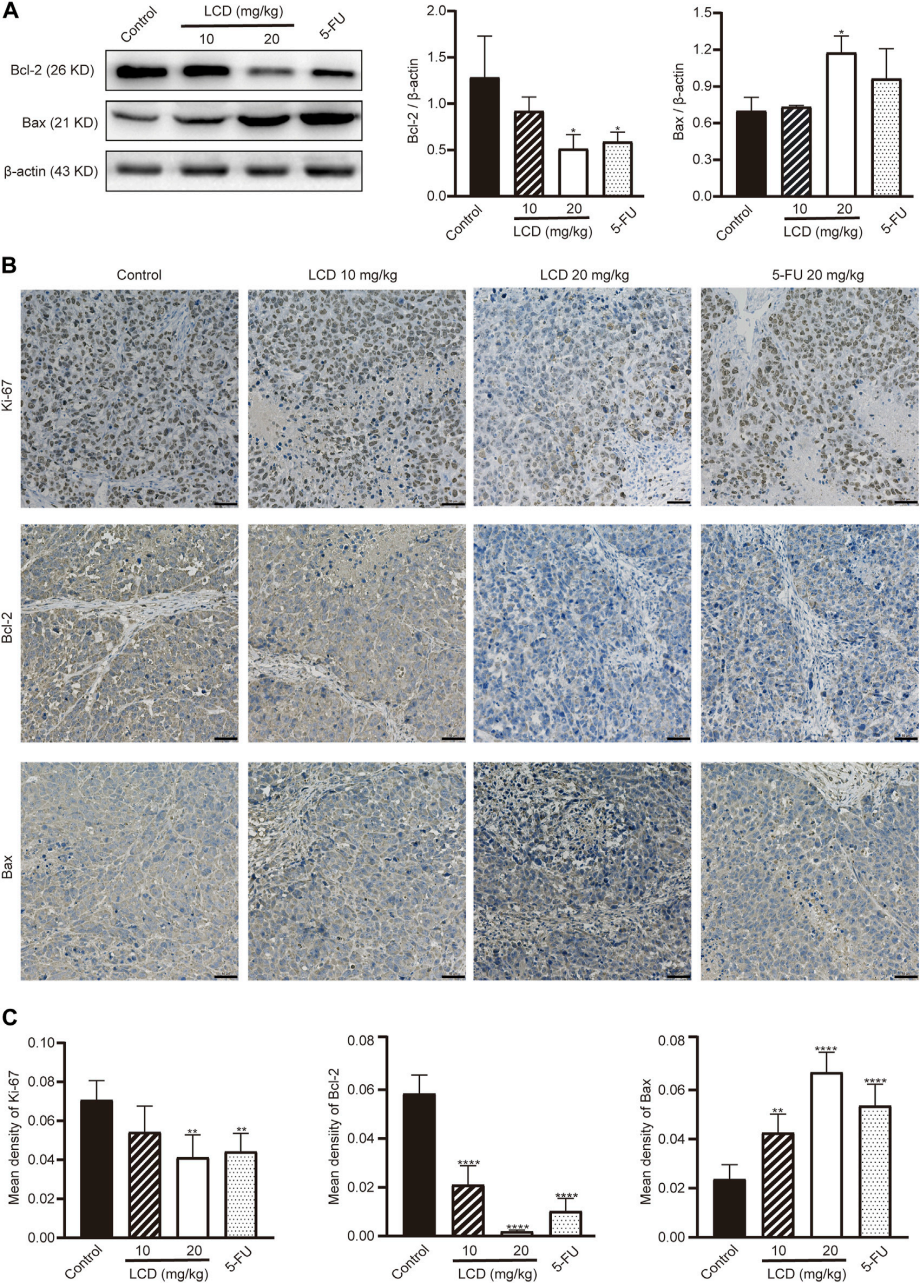
Licoricidin repressed proliferation and induced apoptosis of MGC-803 cells in vivo. **(A)** Expression levels of apoptosis-related proteins (Bcl-2 and Bax) in the xenografted tumor were measured by Western blot assay and normalized by β-actin for quantitative analysis. Independent experiments were repeated three times. **(B)** Immunohistochemistry was used to evaluate the levels of proteins related to proliferation (Ki-67) and apoptosis (Bcl-2 and Bax) in xenograft tumors. **(C)** Mean densities of positively expressed proteins were calculated from five random fields of view by Image-Pro Plus 6.0 software for quantitative analysis. Values are expressed as mean ± SD, *p < 0.05, **p < 0.01, and ****p < 0.0001. LCD, licoricidin. 5-FU, 5-fluorouracil. Scale bar, 50 μm.

Subsequently, we performed Western blot analyses and immunohistochemical staining assays to further detect the expressions of apoptosis-related proteins, as well as proliferation-related protein in tumor tissues. As shown in Figure 5A, the levels of anti-apoptotic protein (Bcl-2) were lower in licoricidin-treated groups than in the control group, assessed by using Western blot analysis. However, levels of pro-apoptotic protein (Bax) showed an opposite trend. As we expected, the expression levels of apoptosis-related proteins reflected by staining density with the immunohistochemistry method (Figures 5B,C) were consistent with Western blot results. In addition, the level of Ki-67, which is a marker protein of proliferation, was obviously reduced by licoricidin using immunohistochemistry assay (Figures 5B,C). Collectively, these data showed that licoricidin suppressed proliferation and induced apoptosis of MGC-803 cells in vivo.

### Exploration for the anti-gastric cancer targets of licoricidin by quantitative proteomics research strategy of TMT labeling

**FIGURE 6:**
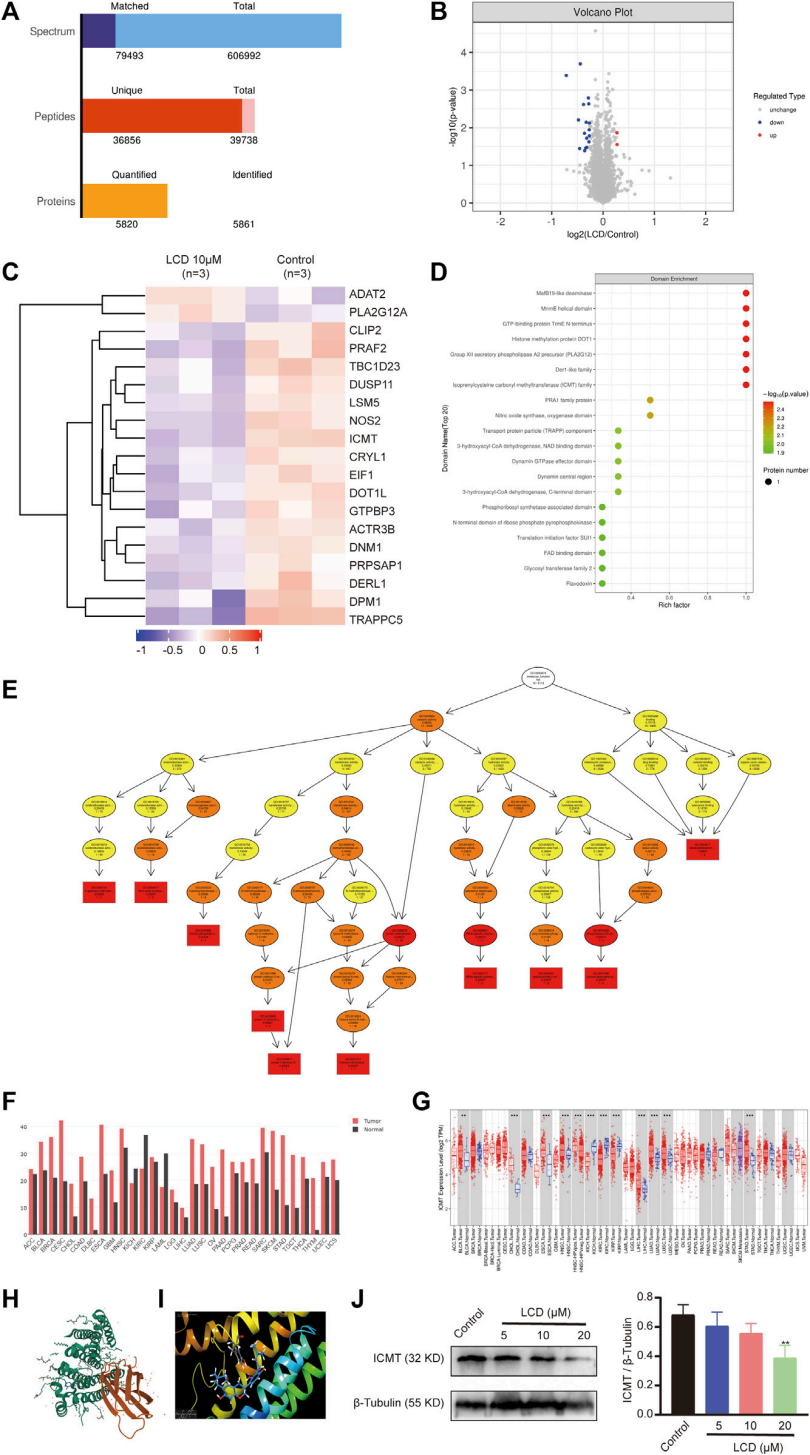
Targets of licoricidin inhibiting the growth of gastric cancer cells were identified by the quantitative proteomics research strategy of TMT labeling. MGC-803 cells were treated with 10 μM of licoricidin or untreated as a control. After protein extraction, mass spectrometry identification, and database search, the differential proteins were screened out. Independent experiments were repeated three times. **(A)** Statistics of the protein identification results. **(B)** Volcano plot of differentially expressed proteins. The significantly downregulated proteins are marked in blue (FC < 0.83 and *p* < 0.05), and strikingly up-regulated proteins are marked in red (FC > 1.2 and *p* < 0.05), while non-differentiated proteins are plotted in gray. **(C)** Hierarchical cluster classified proteins with differential expression and were displayed on the heatmap. The expression levels of significantly different proteins were normalized using the log2 method and revealed in different colors. The red and blue parts represent significantly upregulated and downregulated proteins, respectively. Domain enrichment **(D)** and GO molecular function enrichment **(E)** of differentially expressed proteins were analyzed by Fisher’s exact test. The closer to red color, the higher the enrichment degree. **(F)** Increased or decreased ICMT in different cancers was compared with that of paired normal tissues by gene expression profiling interactive analysis. **(G)** ICMT expression in different tumor types with or without paracancer determined by TIMER. **(H)** Three-dimensional structure of ICMT (PDB code:5V7P) was downloaded from Protein Data Bank (https://www.rcsb.org/3d-view/5V7P). **(I)** Licoricidin bound to the active site (TYR131 residue) of ICMT *via* forming a hydrogen bond in the molecular docking mode. The docking score was −9.165. **(J)** Expression level of ICMT in vitro was demonstrated by Western blot assay and normalized by β-tubulin for quantitative analysis. Independent experiments were repeated three times. Values are expressed as mean ± SD, **p < 0.01 and ***p < 0.001. LCD, licoricidin. 5-FU, 5-fluorouracil.

To search the anti-gastric cancer target of licoricidin, differential expression proteins were screened out using the quantitative proteomics research strategy of TMT labeling. MGC-803 cells were treated with 10 μM of licoricidin for 24 h, and a control group treated by an equal amount of the solvent was set for comparison. A total of 5861 proteins were identified (Figure 6A). Differentially expressed proteins were screened out based on the Fold of Change (FC) expression over 1.2 times and a *p* value <0.05 (paired Student’s *t*-test). As shown in Figures 6B,C, 19 differential proteins were ultimately obtained, including two upregulated proteins (FC > 1.2 and *p* < 0.05) and 17 downregulated proteins (FC < 0.83 and *p* < 0.05). Next, domain enrichment (Figure 6D) analysis and GO functional annotation (Figure 6E) of differentially expressed proteins were performed using Fisher’s exact test. Further screening was carried out by combining the functional enrichment of the differential proteins with the literature and bioinformatics data. The analysis results revealed that ICMT was one of the differential proteins with the highest expression fold and the smallest *p* value (FC = 0.734805227, *p* = 0.000201807). The molecular function of protein C-terminal S-isoprenylcysteine carboxyl O-methyltransferase activity associated with ICMT was significantly changed. Moreover, bioinformatics data from the GEPIA and TIMER database also showed that ICMT functioned as an upregulation protein in gastric cancer (Figures 6F,G). Furthermore, according to the structural characteristics of ICMT protein (Figure 6H), the computational molecular docking model showed that licoricidin could bind to the active site (TYR131 residue) of ICMT by forming a hydrogen bond. The docking score was -9.165, which showed that the docking model was accurate and reliable (Figure 6I). Finally, Western blot assay confirmed that with an increasing dose of licoricidin exposure in MGC-803 cells, the expression level of ICMT was downregulated in a concentration-dependent manner (Figure 6J). Consequently, these results lead us to speculate that ICMT may be the anti-gastric cancer target of licoricidin.

### Licoricidin exerted the anti-gastric cancer effect by blocking the ICMT/Ras signaling pathway in vivo

**FIGURE 7:**
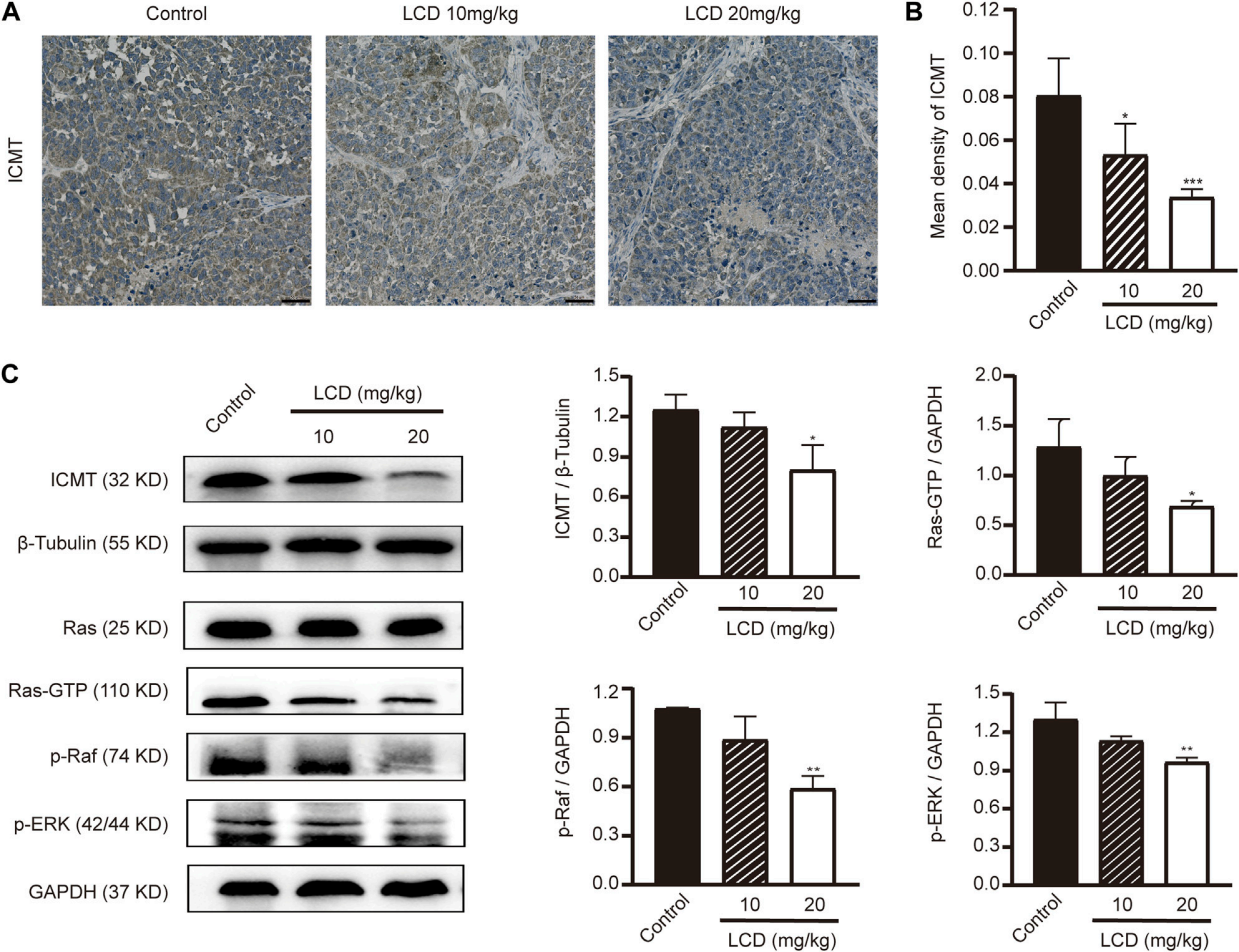
Licoricidin blocked the ICMT/Ras signaling pathway *in vivo*. **(A)** Immunohistochemical staining images of ICMT in xenografted tumors treated with different concentrations of licoricidin. **(B)** Mean densities of positively expressed ICMT protein were calculated from five random fields of view by Image-Pro Plus 6.0 software for quantitative analysis. **(C)** Expression levels of proteins (ICMT, Ras-GTP, p-Raf, and p-Erk) taking part in the ICMT/Ras signaling pathway in xenografted tumors were assessed by Western blot analysis and normalized by reference genes for quantitative analysis. Results are displayed as mean ± SD of three independent experiments. *p < 0.05, **p < 0.01, and ***p < 0.001. LCD, licoricidin. 5-FU, 5-fluorouracil. Scale bar, 50 μm.

To elucidate the profound antineoplastic mechanism of licoricidin against gastric cancer, an immunohistochemistry staining method and Western blot analyses were used to detect the expression level of ICMT in xenografted tumors. With the increasing dose of licoricidin, the degree of staining of ICMT was attenuated (Figures 7A,B), and its expression level was downregulated (Figure 7C). Namely, licoricidin could impair the expression level of ICMT in vivo. The levels of proteins belonging to the Ras signaling pathway were then detected by Western blot assays (Figure 7C). Compared with the control group, active Ras-GTP were significantly reduced in tumors exposed to licoricidin in a dose-dependent manner. Moreover, consistent with the trend of Ras-GTP, the phosphorylation of Raf and Erk in the licoricidin-treated groups was also effectively blocked. It was indicated that the anti-gastric cancer effects of licoricidin were exerted *via* the ICMT/Ras pathway.

## Discussion

Licoricidin is a plant flavonoid extracted from the root of licorice. Previous studies have shown that licoricidin has curative benefits on stomach ulcers (Shibata and Saitoh, 1968) and has noteworthy antibacterial efficacy on *Helicobacter pylori* (Fukai et al., 2002). Moreover, it also possesses remarkable anti-cancer activities against some tumor cells (Park et al., 2010; Park et al., 2016; Ji et al., 2017; Wang et al., 2018; Cevik et al., 2019). In addition, it is protective against the carcinogen MNU (Inami et al., 2017). On the basis of those findings, we first propose and prove that licoricidin has multifaceted anti-cancer activity against gastric cancer. Further studies screen out its target proteins by the quantitative proteomics research strategy of TMT labeling and clarify that licoricidin exerts anti-gastric cancer effects by inhibiting the ICMT/Ras pathway.

First, we demonstrate that licoricidin possesses an attractive anti-cancer property against MGC-803 cells in vitro and in vivo. Although the anti-proliferation activity of licoricidin seemed to be slightly inferior to that of 5-fluorouracil at the same dose in animal experiments, the difference is not significant. In addition, the average body weight of nude mice in the 5-fluorouracil group is always at the lowest level throughout the whole experiment, which may be associated with its toxicity. Compared with the control group, the body weight in the licoricidin-treated groups increases rather than decreasing. This suggests that licoricidin administration could result in a better health status of nude mice with a less toxic effect. Similar results were observed in the study of human colorectal adenocarcinoma (Ji et al., 2017). As for its mechanism, a large body of recent research has indicated that tumor cells are mainly compromised in their ability to exit the cell cycle and consequently continue to divide and grow (Matthews et al., 2022). Therefore, slowing down the process of the cell cycle is an effective measure to inhibit tumor growth (Suski et al., 2021). Our study shows that licoricidin clearly accumulates MGC-803 cells in the G_0_/G_1_ phase. In line with this, the expressions of cyclin D1 and cycle-dependent kinase CDK4 were also significantly downregulated with licoricidin treatment, which promotes the exit of tumor cells from cell cycle and inhibition of cancer proliferation. In addition, the balance of anti-apoptotic *versus* pro-apoptotic Bcl-2 proteins in mammalian cells has been regarded as a cellular rheostat, which manages the threshold to initiate apoptosis (Kalkavan and Green, 2018). In our study, licoricidin breaks the abnormal balance of gastric cancer cells by upregulating Bax and downregulating Bcl-2, thereby leading to the release Cyt-C from the mitochondria into the cytoplasm and to activate Caspase 3. Therefore, licoricidin induces apoptosis of MGC-803 cells through the mitochondrial pathway. Furthermore, Park’s team successively demonstrated that licoricidin could remarkably repress the metastasis and invasion of DU145 prostate cancer cells (Park et al., 2010) and inhibit lung metastasis of 4T1 mammary carcinoma cells (Park et al., 2016). In the present study, we found that licoricidin effectively prevents cells passing through the transwell membranes, reduces the rate of wound healing, and suppresses the activity of matrix metalloproteinase (MMP)-2/9. This suggests that licoricidin might be a potent anti-migration agent for malignant tumors, which is consistent with previous research. Collectively, we prove that licoricidin exerts various anti-tumor activities against gastric cancer.

The interaction of active natural products with intracellular targets is the foundation for producing pharmacological effects. Proteins are major executors of cellular functions, while the targets are proteins that interact with natural products in most cases (Zhou and Xiao, 2017). The quantitative proteomics research strategy of TMT labeling, which is a common high-throughput screening method (Ross et al., 2004; Kalesh et al., 2016), is used to identify the probable intracellular targets of licoricidin against MGC-803 cells. Combined with the functional enrichment of the differential proteins with the literature and bioinformatics data, the ICMT protein is finally distinguished from 19 differential proteins for the following reasons: ICMT is one of the differential proteins with the highest expression fold and the smallest *p* value. The molecular function of protein C-terminal S-isoprenylcysteine carboxyl O-methyltransferase activity that ICMT takes part in is significantly changed in the licoricidin-treated groups. Again, it has been displayed through bioinformatics analysis that ICMT is an upregulation protein in gastric cancer (Figures 6F,G) and that licoricidin could bind to the TYR131 residue of ICMT by forming hydrogen bonds via the computational molecular docking model (Figures 6H,I). As we expected, our study demonstrates that licoricidin reduces the expression level of ICMT by Western blot assay and the immnohistochemistry staining method. Consequently, ICMT is considered to be the most probable target of licoricidin.

ICMT is an exclusive key enzyme that is involved as the catalyst of the carboxymethylation process of C-terminal isoprenylated cysteine of CaaX proteins, including Ras proteins (Winter-Vann and Ca Sey, 2005). This specific methylation event is the last step of post-translational modifications, and it plays a vital role in malignant transformation and tumor maintenance by all Ras isoforms (Lau et al., 2017). Ras proteins belong to the family of small GTPases (Chen et al., 2019) and contain tightly adjusted binary molecular switches, which transition between guanosine diphosphate (GDP)-bound and guanosine triphosphate (GTP)-bound conformations (Simanshu et al., 2017). Once bound to GTP, Ras proteins become active and signal *via* networks that include Raf/Mek/Erk and PI3K/Akt (Mo et al., 2018). It should be noted that about 19% of patients who suffer from cancer harbor Ras mutations (Prior et al., 2020), which are associated with more malignant phenotypes and poorer clinical outcomes (Chen et al., 2021). Previous studies have shown that the loss of ICMT with genetic knockout technology resulted in K-Ras mislocalization in mouse cells (Bergo et al., 2000), and inactivation of ICMT ameliorates malignant phenotypes in a mouse model induced by K-Ras (Wahlstrom et al., 2008). In ovarian cancer cells, ICMT inhibition by siRNA or inhibitor cysmethynil could suppress growth and promote apoptosis. ICMT depletion *via* siRNA significantly reduced the resistance to doxorubicin in HCC cells and inhibited epithelial–mesenchymal transition (EMT) (Xu J. et al, 2019). Meanwhile, 6-C-(E-phenylethenyl) naringenin (6-CEPN), which is a small molecule found in naringenin-fortified fried beef, arrested the cell cycle in the G_1_ phase and induced necrotic cell death and cytoprotective autophagy in colon cancer cells *via* ICMT/RAS signaling pathways (Zhao et al., 2016). In brief, because Ras proteins play an important role in activating cancerous signaling pathways and participate in converting the extracellular environment into intracellular signal transduction cascades, targeting ICMT to block Ras activation may also become a hopeful breakthrough for development of anti-cancer agents (Yang et al., 2017).

In recent years, researchers have detected Ras mutations in gastric cancer (Huang et al., 2016), and data from The Cancer Genome Atlas (TCGA) showed that the K-Ras mutation is the most common type (Chen et al., 2019; Cancer Genome Atlas Research Network, 2014). Peng et al. reported that the total frequency of K-Ras mutation in 126 tissue and nine plasma samples of gastric cancer was 6.67% using Nested and COLD-PCR methods (Peng and Zhao, 2014). Another study has indicated that K-Ras mutation may be involved in the early phase of carcinogenesis of differentiated gastric cancer (Hiyama et al., 2002). As mentioned above, we have shown that licoricidin downregulates the level of ICMT and inhibits the growth of gastric cancer. We further detect the expression levels of downstream signaling molecules of Ras in xenografted tumors to elucidate the profound anti-cancer mechanism of licoricidin. It is well-known that the Raf/Mek/Erk cascade, which is one of the major Ras downstream signaling pathways, is involved in proliferation, apoptosis, cell cycle, invasion, migration, and metastasis of gastric cancer (Magnelli et al., 2020; Zhou et al., 2020) and is considered to be the compelling target for Ras-driven cancer therapy (Tatli and Dinler Doganay, 2021). In this study, in comparison with the control group, levels of active Ras-GTP are significantly reduced in tumors exposed to licoricidin, in a dose-dependent manner. Moreover, along with the inactivation of Ras, the phosphorylation of Raf and Erk in the licoricidin-treated groups is also effectively blocked. This results in the failure of transducing downstream signals and achieving anti-cancer efficiency. Concisely, our work proves that licoricidin exerts promising anti-gastric cancer activities *via* the ICMT/Ras pathway.

In the future, gene knockout technology and molecular docking will be performed to further verify the relationship between licoricidin and ICMT. Moreover, the effects of ICMT on the survival and prognosis of gastric cancer patients will also be explored, which will lay a theoretical foundation for the application of licoricidin in the clinical management of gastric cancer.

## Conclusion

In brief, we first demonstrate that licoricidin possesses various anti-gastric cancer activities in vitro and in vivo, including the inhibition of proliferation, suppression of migration and invasion, induction of apoptosis, and cell cycle arrest. Furthermore, our work also elucidates the mechanism of how licoricidin blocks the ICMT/Ras pathway to achieve anti-gastric cancer efficacy. These results suggest that licoricidin may become a promising candidate agent for the treatment of gastric cancer.

## Data Availability

The data sets presented in this study can be found in online repositories. The names of the repository/repositories and accession number(s) can be found in the article/Supplementary Material.
